# Is adiponectin associated with acute myocardial infarction in Iranian non obese patients?

**DOI:** 10.1186/1476-511X-8-17

**Published:** 2009-05-28

**Authors:** Mohammad Shojaie, Abdoreza Sotoodah, Ghafar Shafaie

**Affiliations:** 1 Cardiology Department, Jahrom Medical University, Jahrom, Iran; 2 Immunology Department, Jahrom Medical University, Jahrom, Iran; 3 Peymanieh Hospital, Jahrom Medical University, Jahrom, Iran

## Abstract

**Backgrounds:**

Adiponectin is an adipose tissue-derived mediator with significant anti-atherogenic properties. A few studies were done in acute phase of myocardial infarction especially in non obese patients. We design a study to investigate the association between adiponectin concentration and acute phase of myocardial infarction in non obese patients.

**Methods:**

This case-control study was done in Paymaneah Hospital (Jahrom, Iran) from Feb 2007 to May 2008. Plasma adiponectin levels were measured in 43 patients with AMI (mean age: 62.7 ± 13.3 years, male: 67.4%) at the first 24 hours of admission and 43 normal controls (mean age: 62.1 ± 12.3 years, male: 55.8%) matched for age, sex and other CAD risk factors.

**Results:**

Adiponectin levels in patients with AMI (3.36 μg/mL) were significantly lower than that of the control group (5.03 μg/mL) (p < 0.0001). Lower adiponectin were independently associated with higher risk of AMI (odds ratio = 8.97; 95% CIs: 2.3–34.5; p = 0.001). Adiponectin levels negatively correlated with triglyceride (r = -0.46, p = 0.002) and total cholesterol (r = -0.32, p = 0.03) in the case group and with body mass index (BMI) in control subjects.

**Conclusion:**

The present study showed that adiponectin was associated with AMI in non obese patients but it is not related to sex, age and other CAD risk factors.

## Introduction

Adiponectin is a new member of adipocyte derived proteins belonging to the soluble defense collagens that produces specifically by adipose tissue and is present in the circulation [1]. Lower levels of plasma adiponectin have been detected in obesity [1], Type 2 diabetes mellitus [2] and coronary artery disease (CAD) [3]. Adiponectin appears to play an important role in glucose and lipid metabolism, vascular biology, and energy homeostasis [4,5] and low adiponectin level has been shown as a risk factor for cardiovascular events [6,7].

Although several studies have been done on adiponectin and CAD, but most of them have been conducted on patients with chronic ischemia and the studies in acute phase of myocardial infarction (MI) especially in developing countries are limited and rare [8-14]. Yet there is controversy about the association of adiponectin and sex [8,11], CAD risk factors [8,11,14] and race [13]. Although adiponectin decreased in both obesity and AMI, some study [9] considered this for explanation of obesity effect on AMI, but with attention to direct effect of adiponectin on atherosclerosis [15], it seems that hypo adiponectinemia in AMI is independent of obesity.

The purpose of present study was to investigate the association between plasma adiponectin levels and AMI in Iranian non obese patients.

## Methods

### Subjects

This case-control study conducted on 43 consecutive patients with acute myocardial infarction (AMI) (as case group) including 29 men and 16 women with the mean age of 62.72 ± 13.4 y/o out of 850 patients who were referred with chest pain to emergency room at Paymaneah Hospital, a referral center affiliated to Jahrom Medical University (Iran) from Feb 2007 to May 2008. We selected all patients after approved diagnosis of AMI and rule out of other patients with exclusion criteria. We also selected 43 individuals with atypical chest pain and normal coronary angiography as control group and matched them with case group for age, sex and other CAD risk factors such as hypertension (HTN), diabetes mellitus (DM), hyperlipidemia (HLP), body mass index (BMI), smoking and renal function.

The study protocol was approved by research ethics committee of Jahrom Medical University and informed consent was obtained from all participants before enrollment.

A questionnaire including information about the past medical and drug history (HTN, HLP, DM, smoking, chronic diseases such as rheumatologic disorder, renal failure and asthma), family history of CAD and demographic information was completed for each subjects.

The exclusion criteria were the history of infectious diseases, collagen vascular disease such as systemic lupus erythematous, scleroderma, rheumatoid arthritis, cirrhosis, hepatitis, renal disease, malignancy, septicemia, angioplasty and stable or unstable angina.

### Definitions

AMI was defined as resting chest pain lasting more than 30 minutes accompanied by ischemic electrocardiographic changes and was confirmed by the presence of total creatine kinase or creatine kinase isoenzyme (CK-MB) fraction levels of more than twice the upper normal limit. We considered ST elevation MI (STEMI) according to ST segment elevation > 1 mm in two neighbor electrocardiographic leads and non ST elevation MI (NSTEMI) without this finding [16]. The absence of any narrowing in coronary artery diameter was considered as normal coronary angiography. Unstable angina was defined as angina pectoris (or equivalent type of ischemic discomfort) with at least one of three features: occurring at rest (or minimal exertion) and usually lasting > 20 minutes (if not interrupted by nitroglycerin administration); being severe and described as frank pain, and of new onset (i.e., within 1 month; and occurring with a crescendo pattern (i.e., more severe, prolonged, or frequent than previously) [17]. Height and weight were measured with light clothes and without shoes using a stadiometer and electronic scale in the cardiac care unit after confirming patient stable condition. Body mass index was calculated [weight (kg)/height (m)^2^] and used as the criteria of overall adiposity. We defined BMI less than 25 as normal, 26–30 as overweight and above 30 as obese [18]. Blood pressure was measured two times in sitting position after 5 min of rest using a mercury sphygmomanometer (Riester, Germany). Hypertension was defined as blood pressure more than 130/85 mmHg or use of any antihypertensive medication [19]. Diabetes mellitus was defined by a physician's diagnosis, a fasting plasma glucose level of ≥ 126 mg/dl or use of diabetes medications [20]. All subjects completed a research questionnaire on smoking habits.

### Laboratory analysis

Fasting levels of plasma total cholesterol, high density lipoprotein cholesterol (HDL), low density lipoprotein cholesterol (LDL), and triglycerides were determined in Research Laboratory of Jahrom Medical University. Total cholesterol and triglyceride levels were measured by enzymatic techniques using an Selectra E biochromatic analyzer. HDL and LDL level was measured after precipitation of the other lipoproteins with heparin and manganese chloride (Human, Germany). Plasma glucose levels were measured by the glucose oxidase method and serum creatinine by the Jaffe reaction method.

Blood samples (5 cc) for adiponectin were obtained by venipuncture from the patients immediately after admission before starting any IV medication by trained staff and for lipid profile and fasting blood sugar at the first 24 hours of AMI after 12 hours of fasting. In control subjects all blood sample were obtained after 12 hours of fasting then plasma was separated and frozen at -70°C for later processing. Then we determined the plasma adiponectin concentration by enzyme linked immunosorbent assay (ELISA) (Biovendor Laboratory Medicine, Inc., Brno, Czech Republic) (*REF: RD195023100*) in patients and control group. This assay measures the total adiponectin that is all molecular forms. The sensitivity and the intra- and interassay coefficients of variation were 0.8 mg/liter, 6% and 7%, respectively.

### Statistical analysis

Statistical analysis was performed by SPSS (version 11.5; SPSS, Inc., Chicago, IL). Data were expressed as mean ± 1 SD. Continuous variables were summarized as mean ± SD and compared using Student's *t*-test. Discrete variables were presented as frequencies and group percentages. Nominal variables were tested with Pearson's χ^2 ^test and Binary variables were tested with the Mann-whitney test. Pearson correlation coefficients were calculated to evaluate unadjusted (univariate) associations between adiponectin and other variables. All tests were two-tailed with a 0.05 type I error rate. Logistic regression models were used to estimate odds ratios (ORs) between three different adiponectin levels and AMI. We considered adiponectin level greater than 5 μg/ml as reference level.

## Results

The demographic and clinical characteristics of the study groups, as well as laboratory variables are shown in Table 1. In patient group 6 cases (14%) had non-ST elevation MI (NSTEMI) and 37 (86%) had ST elevation MI (STEMI).

**Table 1:** Demographic and clinical characteristics of the study groups

Variable	Case group n = 43	Control group n = 43	P-value
Age	62.7 ± 13.3	62.1 ± 12.3	0.84
Male, n (%)	29 (67.4%)	24 (55.8%)	0.27
BMI (kg/m^2^)	22.3 ± 3	23.6 ± 3.15	0.44
Current smoker, n(%)	10 (23.3%)	4 (9.3%)	0.14
HTN, n(%)	9 (20.9%)	14 (32.6%)	0.33
Adiponectin (μg/mL)	3.3 ± 1.7	5 ± 1.5	< 0.001*
Type 1 DM, n (%)	2 (4.7%)	4(9.3%)	0.68
Type 2 DM, n (%)	7(16.3%)	4(9.3%)	0.52
Total Cholesterol (mg/dL)	189.3 ± 45.2	171.8 ± 33	0.05
LDL-C (mg/dL)	113.7 ± 36.6	103.9 ± 28.7	0.13
HDL-C (mg/dL)	46.7 ± 11.4	41.9 ± 9.5	0.05
LDL/HDL ratio	4.23	4.24	0.96
Triglyceride (mg/dL)	146 ± 99.6	144 ± 99.8	0.93
Serum creatinine (mg/dl)	1 ± 0.1	1.1 ± 0.1	0.68

BMI: body mass index, LDL-C: low density lipoprotein- cholesterol, HDL-C: high density lipoprotein- cholesterol, BUN: blood urea nitrogen

Values are presented as mean ± SD or %.

Plasma adiponectin levels in the patients with AMI on admission were significantly lower than those in the control group (3.3 ± 1.7 μg/ml vs. 5 ± 1.5 μg/ml, p < 0.001).

We examined the association between plasma adiponectin levels and selected cardiovascular risk factors among cases and controls. There was a significant negative correlation between plasma adiponectin levels and triglyceride (r = -0.46, p = 0.002) and total cholesterol (r = -0.33, p = 0.03) in the case group as well as BMI in control group (r = -0.44, p = 0.003). No significant correlation was found between HTN, Type 1 DM, Type 2 DM, age, sex, smoking, serum creatinine, LDL and HDL and adiponectin levels (Table 2). There was no significant difference in plasma adiponectin between patients with STEMI and NSTEMI (3.4 ± 1.7 μg/ml vs. 3 ± 1.5 μg/ml, p = 0.49).

**Table 2:** Pearson Correlation Coefficients between Plasma Adiponectin Level and selected variables in the Case and Control groups

**Variable**	**Case group**		**Control group**	
	Correlation	P-value	Correlation	P-value
Age	0.24	0.04*	0.04	0.79
BMI	-0.03	0.86	-0.44	0.003**
Serum creatinine	0.13	0.41	0.27	0.08
Total Cholesterol	-0.33	0.03*	-0.11	0.48
LDL-C	-0.03	0.84	-0.02	0.87
HDL-C	-0.06	0.72	-0.09	0.55
Triglyceride	-0.46	0.002**	-.09	0.57
TC/HDL	-0.22	0.14	-0.40	0.80

*P < 0.05, **P < 0.001

BMI: body mass index, IDDM: insulin dependent diabetes mellitus, NIDDM: non insulin dependent diabetes mellitus, LDL-C: low density lipoprotein- cholesterol, HDL-C: high density lipoprotein- cholesterol, BUN: blood urea nitrogen

Patients were divided into three groups based on adiponectin split level. The results are shown in Table 3. The group with the adiponectin levels greater than 80^th ^percentile (5 μg/mL) was used as the reference. Odds ratio for AMI increased with decreasing adiponectin levels. Lower adiponectin were independently associated with higher risk of AMI (odds ratio = 8.97; 95% CIs: 2.3–34.5; p = 0.001). The greatest increase in AMI risk was seen at adiponectin levels ≤ 3 μg/mL.

**Table 3:** Odds ratio association of different adiponectin levels with acute myocardial infarction (AMI)

Adiponectin levels	Case group (AMI) (n, %)	Control group **(n,%)**	P value	OR (95% CI)
> 5 μg/mL	9 (20.9%)	19 (44.2%)		Reference group (1.00)
3.01–5 μg/mL	17 (39.5.6%)	20 (46.5%)	0.26	1.97 (0.65–4.99)
≤ 3 μg/mL	17 (39.5%)	4(9.3%)	0.001*	8.97 (2.3–34.5)

CI: Confidence Interval, OR: odds ratio

*p < 0.001 significant with single logistic regression analysis

## Discussion

In the present case-control study, in line with previous studies [7-10,21], we found that lower plasma adiponectin levels was associated with AMI among Iranian non obese patients independent of traditional cardiovascular risk factors. Of note, the relationship was also independent of hypertension, diabetes, age, sex, BMI and smoking; the factors that were closely related to adiponectin levels in previous studies [10].

Cavusoglu et al. [22] showed that high baseline plasma adiponectin levels are independently associated with an increased risk of both death and MI at 2-year follow-up in a cohort of men with stable angina, unstable angina, and non ST elevation MI referred for coronary angiography. Our study also showed significant association between adiponectin levels in patients with ST elevation MI and non-ST elevation MI in spite of their different pathophysiology [23,24]. These findings indicated that adiponectin may be directly involved in the pathophysiology of atherosclerosis and thrombosis at the vascular wall level.

The exact mechanism of adiponectin decrease immediately after the onset of AMI remains unknown. Although some authors [25] believed that the changed adiponectin levels are associated with presence of risk factors of metabolic syndrome but our findings in line of other studies emphasis that its deficiency may lead to thrombus formation and platelet aggregation [15]. Plasma adiponectin may decrease as a result of rupture of coronary plaques. Adiponectin has been detected in the injured vessels but not in the intact ones in humans and rodents [26,27]. Although it seems that low adiponctin in AMI is secondary to plaque rupture and inflammation but if according to previous cohort studies [14,22,28] adiponectin consider as risk factor for coronary artery disease, measurement of adiponectin levels may be helpful in the stratification of patients at risk of AMI. In our study the greatest increase in MI risk occurred at adiponectin levels ≤ 3 μg/mL in contrast to the Wolk et al. study [9] that this rise occurred at adiponectin levels ≤ 5.5 μg/mL. This difference may be due to exclusion of the patients with major CAD risk factors in Wolk et al. study that were not excluded from us. Our present data, consistent with those of other authors [8,11,14,22,25] showed that adiponectin levels was negatively correlated with triglycerides in patients and with BMI only in the control subjects, but in contrast to the others we found negative correlation with total cholesterol in patients and any correlation with other CAD risk factors such as smoking[14], age, DM, HTN, HDL and LDL.

About non significant correlation between plasma adiponectin levels and BMI in case group, it should be noted that in the most of previous studies [8,11,25,29] there were significant but weak correlation (r = -0.18 to -0.33) between them, So, in confirming Pliz et al opinion [11], it should be noted that the adiponectin levels may be more associated with blood lipid profile and insulin resistance instead of obesity. Also this difference may be due to reduction of adiponectin secondary to acute MI.

Most studies [8,10-12,14] showed higher adiponectin levels in women in comparison to men but our results showed no statistically significant difference in adiponectin levels between men and women. Nakamura et al. [8] found similar results but they believed that solid phase ELISA was the reason for this difference, because this assay cannot distinguish between the lower weight trimer-dimer forms of adiponectin and the high molecular weight complexes; one of the factors associated with sex difference. We measured all forms of adiponectin and this difference in our study is not related to this reason. It may be due to less obesity in our subjects and/or mismatch between BMI in men and women in other studies.

Another remarkable clinical finding in our study was that plasma adiponectin levels in our study were much lower than those of other studies [7-9,14] such that this level in our normal subjects was approximately equal to that of patients with AMI. This may be due to lower BMI in our subjects, high carbohydrate diet [30] or race differences.

Kanaya et al. [13] showed that Whites had higher median adiponectin levels than Blacks and high circulating levels of adiponectin were associated with a higher risk of CAD in older Blacks. Significant differences in our results indicate that before application of adiponectin in clinical setting, further prospective and cohort studies in other countries and races are required to confirm our results.

Although some studies [30,31] showed increased adiponectin level in patients with renal dysfunction but our study showed as the same of Shibata et al. [32] that there is no association between adiponectin and renal function in atherosclerotic patients with normal renal function.

### Study limitations

Serum adiponectin levels were measured in a single sample, which might limited the observed associations. Weak or any correlation may be due to small number of participants in this study. We didn't study the association of adiponectin concentration with amount of smoking, diet and previous medication because of limited data. Some authors such as Inoue et al. [33] suggest that measurement of high molecular weight (HMW) adiponectin alone may be a clinically useful marker of CAD but we measured only total form of adiponectin. Although abdominal obesity can give better insight than BMI, but because some studies use only BMI, for comparison we only used this parameter too.

## Conclusion

Lower plasma adiponectin levels are associated with AMI in Iranian non obese patients, independent of other traditional cardiovascular risk factors. Our findings showed although obesity is a reducing factor of adiponectin but this decreased in AMI is not related only to obesity.

## Competing interests

The authors declare that they have no competing interests.

## Authors' contributions

MS had substantial contributions to conception and design and interpretation of data and writing the manuscript. AS carried out the biochemical analysis. GS had contributions to data analysis. All authors read and approved the final manuscript.
